# Cabozantinib and dastinib exert anti-tumor activity in alveolar soft part sarcoma

**DOI:** 10.1371/journal.pone.0185321

**Published:** 2017-09-25

**Authors:** Kenta Mukaihara, Yu Tanabe, Daisuke Kubota, Keisuke Akaike, Takuo Hayashi, Kaoru Mogushi, Masaki Hosoya, Shingo Sato, Eisuke Kobayashi, Taketo Okubo, Youngji Kim, Shinji Kohsaka, Tsuyoshi Saito, Kazuo Kaneko, Yoshiyuki Suehara

**Affiliations:** 1 Department of Orthopedic Surgery, Juntendo University School of Medicine, Tokyo, Japan; 2 Department of Human Pathology, Juntendo University School of Medicine, Tokyo, Japan; 3 Center for Genomic and Regenerative Medicine, Juntendo University School of Medicine, Tokyo, Japan; 4 Department of Physiology and Cell Biology, Tokyo Medical and Dental University Graduate School and Faculty of Medicine, Tokyo, Japan; 5 Division of Musculoskeletal Oncology, National Cancer Center Hospital, Tokyo, Japan; 6 Department of Medical Genomics, Graduate School of Medicine, The University of Tokyo, Tokyo, Japan; National Institute of Health, UNITED STATES

## Abstract

**Background:**

Alveolar soft part sarcoma (ASPS) is an extremely rare metastatic soft tissue tumor with a poor prognosis for which no effective systemic therapies have yet been established. Therefore, the development of novel effective treatment approaches is required. Tyrosine kinases (TKs) are being increasingly used as therapeutic targets in a variety of cancers. The purpose of this study was to identify novel therapeutic target TKs and to clarify the efficacy of TK inhibitors (TKIs) in the treatment of ASPS.

**Experimental design:**

To identify novel therapeutic target TKs in ASPS, we evaluated the antitumor effects and kinase activity of three TKIs (pazopanib, dasatinib, and cabozantinib) against ASPS cells using an *in vitro* assay. Based on these results, we then investigated the phosphorylation activities of the identified targets using western blotting, in addition to examining antitumor activity through *in vivo* assays of several TKIs to determine both the efficacy of these substances and accurate targets.

**Results:**

In cell proliferation and invasion assays using pazopanib, cabozantinib, and dasatinib, all three TKIs inhibited the cell growth in ASPS cells. Statistical analyses of the cell proliferation and invasion assays revealed that dasatinib had a significant inhibitory effect in cell proliferation assays, and cabozantinib exhibited marked inhibitory effects on cellular functions in both assays. Through western blotting, we also confirmed that cabozantinib inhibited c-MET phosphorylation and dasatinib inhibited SRC phosphorylation in dose-dependent fashion. Mice that received cabozantinib and dasatinib had significantly smaller tumor volumes than control animals, demonstrating the *in vivo* antitumor activity of, these substances.

**Conclusions:**

Our findings suggest that cabozantinib and dasatinib may be more effective than pazopanib against ASPS cells. These *in vitro* and *in vivo* data suggest that c-MET may be a potential therapeutic target in ASPS, and cabozantinib may be a particularly useful therapeutic option for patients with ASPS, including those with pazopanib-resistant ASPS.

## Introduction

Alveolar soft part sarcoma (ASPS) is an extremely rare soft tissue tumor that generally occurs in the extremities of young adults [1–3]. ASPS has a high frequency of metastases to the brain, lungs, and bones [1–3]. The rate of metastatic disease at the time of diagnosis is reported to be 20%–65% [1–3]. Despite the relatively indolent clinical course of the disease, its prognosis remains poor owing to the high rate of metastasis, and the 10-year survival rate is 48% [4]. Surgical resection is the only known curative therapy for localized disease, as ASPS has been shown to be resistant to conventional chemotherapy and radiation [5, 6]. Most patients with unresectable metastatic ASPS cannot be cured. Novel systemic therapeutic options are therefore needed, particularly for advanced cases.

The overall approach to the treatment of cancer is currently undergoing a drastic shift, from the existing broadly toxic chemotherapeutic agents to molecular-targeted therapy [7]. Tyrosine kinases (TKs) are attractive as therapeutic targets, as aberrant signaling via TKs plays an important role in the progression of numerous human cancers, despite the fact that TKs account for less than 1% of all protein kinases [8]. Currently, 90 unique TKs have been identified in the human genome: 58 receptor-type TKs and 32 nonreceptor-type TKs [9]. TKs are the most common and successful targets used in rational oncology drug discovery, as represented by imatinib for chronic myelogenous leukemia and gastrointestinal stromal tumors, trastuzumab for breast cancers, and gefitinib for lung cancers [10–14].

ASPS is associated with a characteristic chromosomal translocation: der(17)t(X;17)(p11;q25), resulting in the *ASPL-TFE3* fusion gene, which is critical for tumor development [4]. Functional data link the characteristic translocation (*ASPL-TFE3*) in ASPS to the *ASPL-TFE3* fusion gene, which is shown to upregulate the expression of the c-MET receptor TK [15–20]. Furthermore, a recent gene expression profiling study in ASPS revealed that certain TKs (c-MET and VEGFR) were expressed in ASPS and related to the malignant features of the tumor cells [15–19, 21, 22]. Therefore, these TKs are expected to be potential therapeutic targets in ASPS.

Pazopanib was recently approved by the U.S. Food and Drug Administration (FDA) for the clinical treatment of advanced soft tissue sarcoma. Pazopanib is a novel TK inhibitor (TKI) that targets PDGFR, VEGFR, and c-kit [23–26]. Some published studies have reported acceptable response rates of ASPS to treatment with pazopanib, despite the low response rates observed with most types of soft tissue sarcoma (STS) [23–26]. However, the mechanisms of action of pazopanib and the responses to the drug in ASPS tumor cell lines have not yet been explored. In addition, despite the reported findings of some clinical trials in ASPS patients using TKIs (including tivantinib, bevacizumab, sunitinib, and cediranib) that several TKIs have an antitumor effect, the effects of these agents in *in vitro* assays have yet to be demonstrated [27–32]. At present, the effects of TKIs such as cabozantinib, which targets c-MET, VEGFR2, FLT3, c-kit and RET; and dasatinib, which targets SRC and ABL, have not been investigated [33–36].

In the present study, to identify novel therapeutic targets for TKIs in ASPS, we investigated the *in vitro* antitumor activity of the TKIs pazopanib, cabozantinib, and dasatinib, which were selected based on previous findings in an ASPS human cell line [15–19, 21, 22, 37]. After examining the *in vitro* antitumor activities of these agents, in order to elucidate the functions of the drug effects *in vitro* and the antitumor activity *in vivo*, we investigated the phosphorylation activities of the targetable proteins and the antitumor activity through *in vitro* assays using these TKIs.

## Materials and methods

### Cell line

The ASPS cell line ASPS-KY was kindly provided by the Kanagawa Cancer Center [38–40]. The *ASPL-TEF3* fusion gene was confirmed by reverse transcription-polymerase chain reaction (RT-PCR) (S1 Fig). ASPS-KY cells were grown as monolayers in DMEM (Sigma-Aldrich, St. Louis, MO, USA) supplemented with 10% FBS (Life Technologies, Bethesda, MD, USA) and maintained at 37°C in a 5% CO_2_ incubator.

### TKIs

Pazopanib (GW786034) and dasatinib (BMS-354825) and cediranib (S1017) were purchased from Selleck Chemicals (Houston, TX, USA); cabozantinib (XL-184) was obtained from ChemScene (Monmouth Junction, NJ, USA); and sunitinib (PZ0012) was purchased from Sigma-Aldrich. All inhibitors were dissolved in DMSO for the *in vitro* studies.

### Cell proliferation assay

ASPS-KY cells were seeded in 96-well plates at 3000 cells/well and allowed to adhere overnight. The next day, different concentrations of TKIs or DMSO (as a vehicle control) were added to each well, and the cells were incubated for another 96 h. After incubation, the Cell Counting Kit-8 (Dojindo Laboratories, Kumamoto, Japan) reagents were added to each well. After incubation for 2 h, cell proliferation was assessed by measuring the absorbance at 450 nm with a microplate reader (Tecan Safire, Tecan Group AD, Manndorf, Switzerland).

### Matrigel invasion assay

Invasion assays were performed using a 24-well BD BioCoat Matrigel Invasion Chamber (BD Biosciences, Bedford, MA, USA) in accordance with the manufacturer’s protocol. A total of 2×10^5^ ASPS-KY cells in 0.2% FBS-containing medium were placed in the upper wells of the chamber. The lower chamber contained medium with 10% FBS. The chambers were treated with DMSO as a control or with different doses of TKIs. After 48 h of incubation, non-invading cells were removed from the upper chamber with cotton swabs. The invaded cells were stained and visualized using a Diff-Quick reagent (Sysmex International Reagents, Kobe, Japan). The cells were digitally photographed (×200 magnification) and counted. The invasion index was calculated by comparing the percent invasion of the untreated cells with that of those treated with a TKI.

### Western blot analyses of protein phosphorylation

ASPS-KY cells were starved in serum-free medium overnight. The cells were then incubated for 2 h with increasing concentrations of TKIs. Only the cells that were scheduled to be treated with cabozantinib were further stimulated with either 20 ng/mL HGF (R&D Systems, Minneapolis, MN, USA) or 50 ng/mL VEGFA (Cell Signaling Technologies, Beverly, MA, USA) for 10 min.

The proteins were separated via SDS-PAGE and transferred to nitrocellulose membranes, which were incubated with the following antibodies: antibodies against MET, p-(Tyr1234/1235)-MET, SRC, p-(Tyr416)-SRC, AKT, p-(Ser473)-AKT, ERK1/2, p-(Thr202/Tyr204)-ERK1/2, FAK, p-(Tyr397)-FAK (all Cell Signaling Technologies), VEGFR2 (Cell Signaling Technologies), p-VEGFR2 (Cell Signaling Technologies) and GAPDH (Santa Cruz Biotechnology, Santa Cruz, CA, USA). After incubation with the primary antibodies, the membranes were washed three times with Tris-EDTA buffer and then incubated with horseradish peroxidase-conjugated secondary antibodies (GE Healthcare Biosciences, Piscataway, NJ, USA). The expression of each protein was detected using an enhanced chemiluminescence system (ECL Prime; GE Healthcare Biosciences) and the ImageQuant LAS 4000 (GE Healthcare Biosciences).

### *In vivo* animal models

All animal studies were conducted in accordance with protocols approved by the Institutional Animal Care and Use Committee of Juntendo University. All animal experiments were also performed in accordance with the Fundamental Guidelines and Basic Policies for Proper Conduct of Animal Experiments of Juntendo University. Mice were housed in individually ventilated cages (IVC, Tecniplast, SPA, Varese, Italy) in a specific pathogen-free facility on a 12 h light/dark cycle with *ad libitum* access to sterilized standard rodent diet and water. For euthanasia of mice, we used inhalation of carbon dioxide gas. Prior to injection, ASPS cells (5 × 10^6^) were mixed in PBS with Matrigel (BD Biosciences) at a 1:1 ratio. The cell suspension was injected subcutaneously (100 μL/mouse) onto the back of 5- to 6-week-old female BALB/c nude mice (CREATECH, Shizuoka, Japan). When tumors reached approximately 50 to 150 mm^3^ in size, the mice were randomized into 3 groups (n = 4 per group) that orally received vehicle, 15 mg/kg of dasatinib or cabozantinib, or 30 mg/kg of dasatinib or cabozantinib (1 group each). All treatments were administered for 4 weeks (5 days/week). Tumor volumes and body weights were measured every 7 days starting with the first day of treatment. The mice were sacrificed after 4 weeks of treatment. Solid tumors on the mice were ultimately resected.

## Results

### *In vitro* antitumor activity of pazopanib, cabozantinib, and dasatinib in ASPS cells

**Fig 1:**
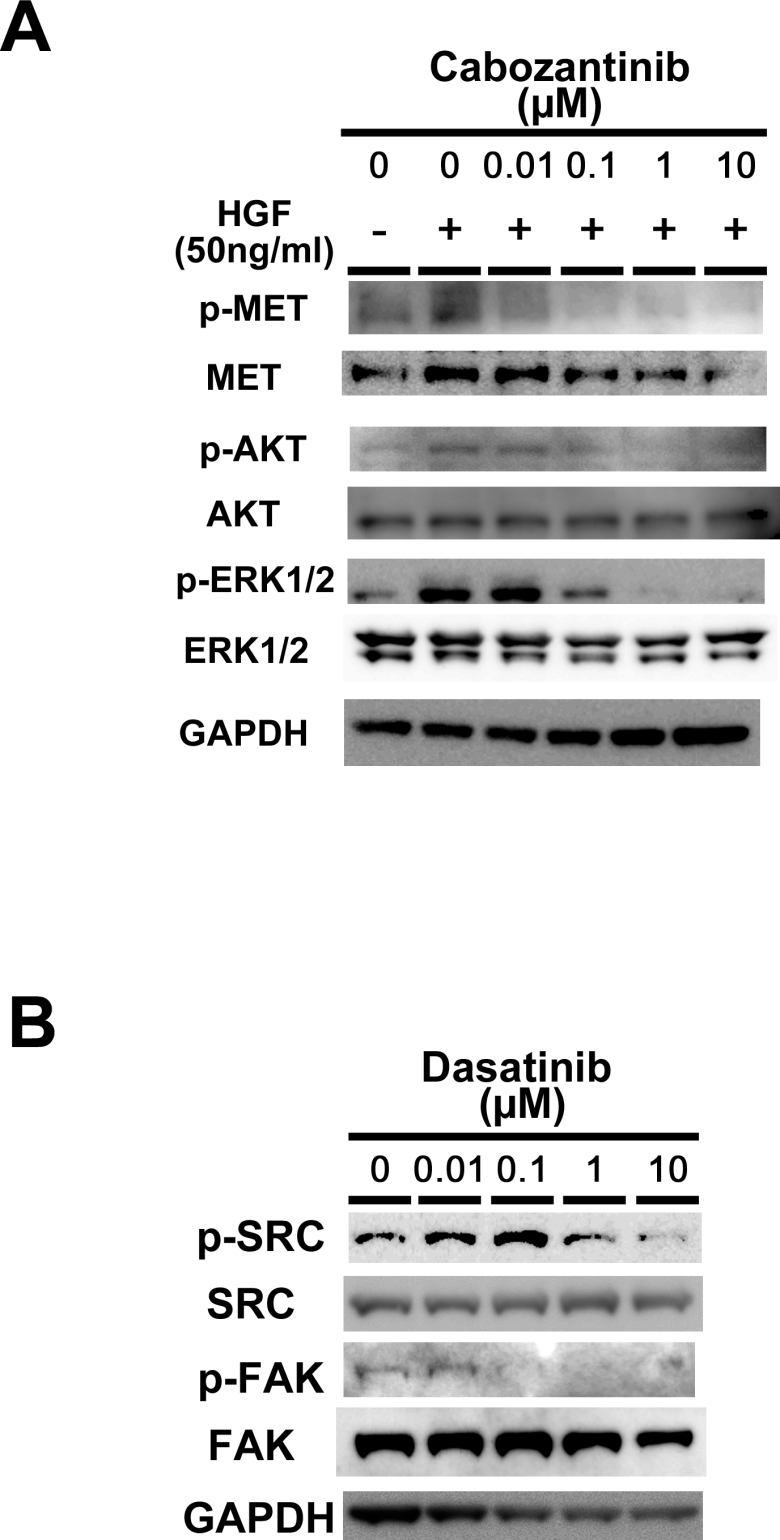
The results of *in vitro* proliferation and invasion assays in ASPS cells treated with three TKIs (pazopanib: A and B, cabozantinib: C and D, dasatinib: E and F). Cell proliferation assays were performed using each of the TKIs (pazopanib: A, cabozantinib: C, dasatinib: E). In these assays, pazopanib had no significant effect on cell proliferation (A), while cabozantinib and dasatinib led to significant inhibition of cell growth compared to control cells (C and E). Cell invasion assays were also performed using each of the TKIs (pazopanib: B, cabozantinib: D, dasatinib: F). These invasion assays revealed that pazopanib and dasatinib had no significant effect on cell invasion (B and F), although cabozantinib significantly inhibited cell invasion compared to control cells (D) (ns: not significant, **: *p* < 0.01, *: *p* < 0.05 by Student's *t*-test).

We used the TKIs pazopanib, cabozantinib, and dasatinib based on the findings of previous studies [15–19, 21, 22, 37]. To evaluate whether or not pazopanib, cabozantinib, and dasatinib inhibited various cellular functions in ASPS cells, we performed *in vitro* proliferation and invasion assays (Fig 1A–1F). In the cell proliferation assays using the ASPS cell line, pazopanib, cabozantinib, and dasatinib all inhibited ASPS cell growth in a concentration-dependent manner (Fig 1A, 1C and 1E). Surprisingly, both cabozantinib and dasatinib showed more than 60% growth inhibition at 40 μM and 10 μM, respectively, while pazopanib demonstrated less than 30% growth inhibition at 100 μM (Fig 1A, 1C and 1E). In the cell invasion assays using the ASPS cell line, treatment with cabozantinib resulted in a decrease in cell invasion by about 53% at 10 μM; however, neither treatment with pazopanib nor dasatinib showed a significant inhibitory effect on cell invasion (Fig 1B, 1D and 1F). Based on these results, we concluded that cabozantinib and dasatinib had more potent effects than pazopanib in regard to inhibiting the cellular functions in ASPS cells.

### Effect of cabozantinib on c-Met and VEGFR2 activation

**Fig 2:**
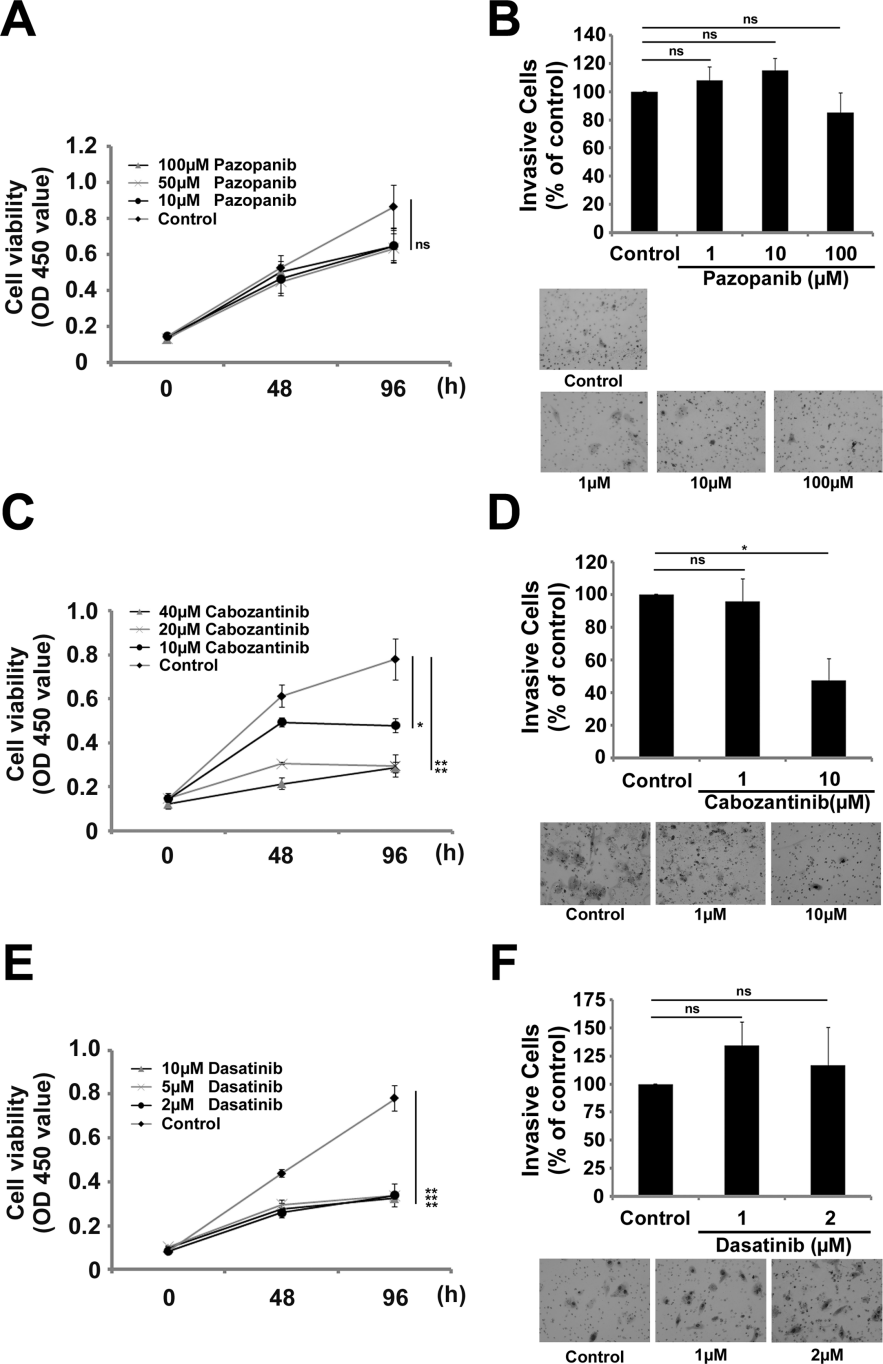
Effects of TKIs on tyrosine phosphorylation and a TK expression analysis by western blot in an ASPS cell line. A: TK phosphorylation assays demonstrated that c-MET stimulated by HGF was inhibited by cabozantinib at increasing concentrations in ASPS cells. Regarding downstream activation of AKT and ERK, cabozantinib inhibited dose-dependent dephosphorylation of p-AKT and p-ERK1/2. B: In the TK phosphorylation assay of SRC using dasatinib, SRC was inhibited by dasatinib. FAK phosphorylation was inhibited by dasatinib at nanomolar concentrations.

In our cell proliferation assays using cabozaninib and targetable genes of cabozantinib, we investigated whether or not cabozantinib affects c-MET phosphorylation. Treatment with cabozantinib resulted in complete inhibition of the c-MET phosphorylation stimulated by HGF at nanomolar concentrations (Fig 2A). This finding suggests that the antitumor activity of cabozantinib may be mediated by c-MET inhibition. We further investigated the downstream activation of AKT and ERK. Dose-dependent dephosphorylation of p-AKT and p-ERK1/2 were observed in ASPS cells at micromolar concentrations of cabozantinib (Fig 2A). These findings suggest that cabozantinib may have potential as a therapeutic agent in the treatment of ASPS and that c-MET may be a good therapeutic target in ASPS. With respect to p-VEGFR2, under VEGFA stimulation, we also confirmed that the expression of VEGFR2 was inhibited in a dose-dependent fashion by cabozantinib (S2 Fig).

### Effect of dasatinib on SRC activation

In our cell proliferation assays using dasatinib and targetable genes of dasatinib, we investigated whether or not dasatinib affects SRC phosphorylation. Treatment with dasatinib resulted in a tendency toward reduction of SRC phosphorylation (Fig 2B). Furthermore, we found that FAK phosphorylation was inhibited by dasatinib at nanomolar concentrations (Fig 2B). These results suggest that the antitumor activity of dasatinib might be mediated by SRC inhibition. Based on these findings, dasatinib may have potential as a therapeutic agent in the treatment of ASPS, and SRC may be a good therapeutic target in ASPS.

### Effect of cabozantinib and dasatinib on the growth of ASPS cells *in vivo*

**Fig 3:**
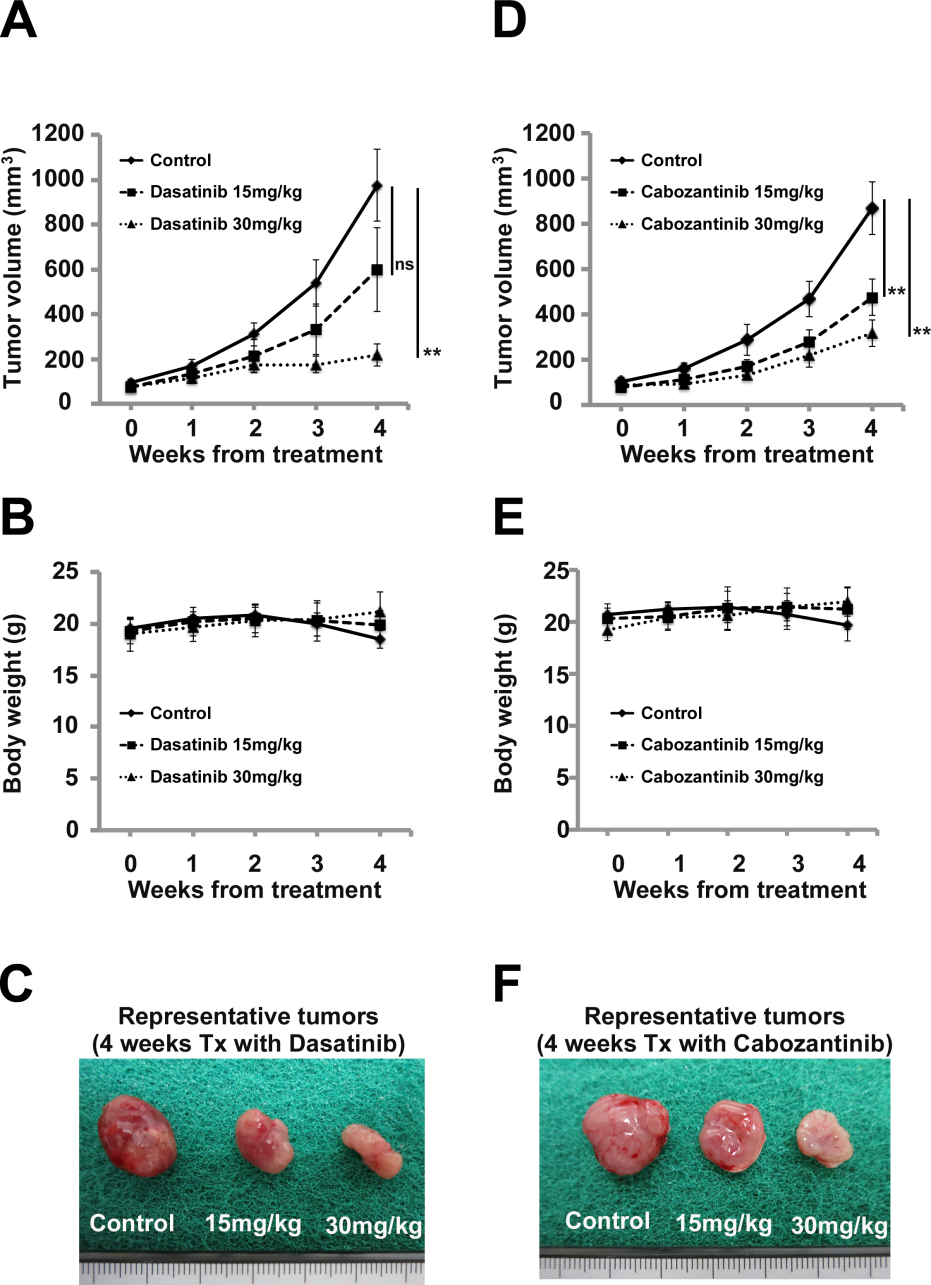
Both cabozantinib and dasatinib significantly inhibited tumor growth of ASPS cells *in vivo*. (A) (D) Calculated tumor volume of ASPS-KY xenografts in BALB/c nude mice treated with vehicle or dasatinib, or cabozantinib, respectively, as measured by digital Vernier calipers is shown (ns: not significant, **: *p* < 0.01 by Student's *t*-test). (B) (E) Weight of mice treated with vehicle or dasatinib or cabozantinib measured every week for 4 weeks is shown. There were no differences in the weight of the mice between the drug treatment groups and vehicle groups. (C) (F) Representative resected tumors from vehicle-treated mice (right), 15 mg/kg dasatinib- or cabozantinib-treated mice (middle) and 30 mg/kg dasatinib- or cabozantinib-treated mice (left) after 4 weeks’ administration show the difference in tumor size. These results revealed significant associations between treatment and tumor growth.

To evaluate the *in vivo* antitumor efficacy of cabozantinib and dasatinib in ASPS cells, BALB/c nude mice inoculated subcutaneously with ASPS cells were treated with daily oral cabozantinib (15 or 30 mg/kg) or dasatinib (15 or 30 mg/kg). We measured the tumor volumes every 7 days. In the *in vivo* assays, the mice that received cabozantinib had smaller tumor volumes than the control animals at week 4, as did the mice that received dasatinib (Fig 3A and 3D).

## Discussion

As ASPS is an extremely rare malignant tumor with a poor prognosis, the identification of potential therapeutic targets is necessary [1–3]. TKs are critical therapeutic targets in various cancers, and the utilization of several TK targets has dramatically changed cancer treatment strategies, such as the application of imatinib for chronic myelogenous leukemia and gastrointestinal stromal tumors, trastuzumab for breast cancers, and gefitinib for lung cancers [10–14]. Pazopanib was recently approved by the U.S. FDA for the clinical treatment of advanced STS. Pazopanib is a novel TKI that targets PDGFR, VEGFR, and c-kit [23, 25]. Some studies have reported acceptable response rates of ASPS to pazopanib treatment despite the low response rates of most types of STS [24–26]. Nakamura et al. revealed that 4 of 12 (33.3%) cases of ASPS achieved partial response (PR), while only 13 of 156 (8.3%) cases consisting of several histological types of STS achieved PR with pazopanib treatment [25]. Other case reports have also described the benefits of treatment with pazopanib in ASPS [24, 26].

In the present study, pazopanib inhibited cell growth in ASPS cells. In statistical analyses of cell proliferation and invasion, pazopanib showed an inhibitory tendency in both assays. Based on gene expression profiles in previous studies, ASPS shows high expression of VEGF and VEGFR [15, 16, 19]. Therefore, pazopanib might inhibit cell growth in ASPS through both VEGF and VEGFR. Surprisingly, *in vitro* studies of pazopanib, cabozantinib, and dasatinib have found that both cabozantinib and dasatinib showed significant inhibitory effects on ASPS despite pazopanib’s lack of significant suppression of cell growth. Based on ASPS gene expression profiles and the TK targets of cabozantinib and dasatinib described in previous studies, we verified the functional effects of cabozantinib against c-MET and dasatinib against SRC [15–19, 21, 22, 37]. We found that cabozantinib completely inhibited c-MET phosphorylation and that dasatinib inhibited Src phosphorylation in ASPS cells. Given these findings, we believe that cabozantinib and dasatinib may suppress ASPS cell growth.

Cabozantinib/XL184 is a small-molecule inhibitor that simultaneously targets c-MET, VEGFR2, FLT3, c-Kit, and RET [34, 36, 41]. In our study, treatment of ASPS cells with cabozantinib resulted in marked inhibitory effects on cell proliferation and invasion. Cabozantinib also inhibited c-MET phosphorylation in a concentration-dependent manner [34, 36]. MET is a gene encoding the TK receptor and proto-oncogene c-MET [34, 36]. Several studies have reported that c-MET is overexpressed in ASPS [15–19, 21, 22], and ASPS samples have shown c-Met activation [20, 42]. Functional data link the characteristic translocation (*ASPL-TFE3*) in ASPS to the *ASPL-TFE3* fusion gene, which is shown to upregulate the expression of the c-MET receptor TK [15–19, 21, 22] Furthermore, MET has been revealed to be a direct transcriptional target of *ASPL-TFE3*. In addition, *ASPL-TFE3* binds to strongly activates the MET promoter [15–19, 21, 22]. c-MET is activated by HGF, resulting in autophosphorylation of the receptor [20, 43]. Aberrant activation of HGF/MET signaling is involved in core oncogenic phenotypes, such as uncontrolled cell proliferation, invasion, and metastasis [20, 43]. These findings are consistent with the results of our study. These present and previous data suggest that cabozantinib may regulate *ASPL-TFE3* via the MET pathway. Regarding clinical trials related to targeting c-MET, a phase II clinical trial of tivantinib (ARQ 197) for the treatment of patients with ASPS reported progressive disease in 19% and stable disease in 78% of the 27 patients in whom efficacy could be evaluated [27]. However, disease stabilization in a single-arm study of tivantinib in a population with an indolent disease does not meet the criteria for demonstrating clinical activity [27]. In addition, recent studies have indicated that tivantinib has additional mechanisms of action, other than MET, that may account for its antitumor activity [44–46]. Clinical trials using tivantinib, therefore, have not yet clarified the potential anti-tumor activity and feasibility of the MET inhibitor in ASPS. Our findings may therefore suggest the need for a clinical re-trial using MET inhibitor in ASPS. In addition, the present and previous findings may also contribute to the development of cabozantinib as a potent drug for the treatment of ASPS and suggest that c-MET may be a novel therapeutic target in ASPS.

Dasatinib is a Bcr-Abl TKI and SRC family TKI [33, 35] that has been approved for chronic myelogenous leukemia and Philadelphia chromosome-positive acute lymphoblastic leukemia [33, 35]. It is also being evaluated for its utility in several other cancers, including advanced prostate cancer [33, 35]. *In vitro* assays against ASPS cells showed that cell growth was significantly suppressed in the dasatinib-treated cells compared with the control cells. Furthermore, dasatinib also inhibited src phosphorylation in a concentration-dependent manner and demonstrated positive results in *in vivo* assays. Given these findings, we suspected that dasatinib might suppress growth, and therefore might be a potentially effective candidate drug for ASPS. Regarding suppression of the tumor function by dasatinib, in function studies of ASPS, a genome-wide location analysis of ASPS tumor samples and cell lines expressing *ASPSCR1–TFE3* defined a subset of approximate 400 genes as putative regulated direct targets of *ASPSCR1–TFE3*, including c-MET [17]. In validating the approach to identify genuine *ASPSCR1–TFE3* target genes, two upregulated genes (*CYP17A1* and *UPP1*) were shown by multiple lines of evidence to be direct, endogenous targets of transactivation by *ASPSCR1–TFE3* [17]. In gene expression studies of SRC transformed by several cells, the genes *IL8/CEF4*, *VIP*, *HMOX1*, *PLCPI*, and *UPP1* were identified as signature SRC aggressive tumor genes [17, 37]. Furthermore, a bioinformatic analysis of gene expression regarding ASPL-TFE3 fusion in ASPS identified several potential therapeutic targets [15]. That study identified CCL4 and CDC6, which are located in the TFE3 neighboring cytogenetic band at chr17q21 as possible targets of treatment. CCL4 was shown to have cellular functions related to protein tyrosine kinase activity linked to this chemokine family, including the SRC kinase Lyn, PI3K, focal adhesion related kinase Pyk2, and members of the MAPK family. Therefore, the study supposed that associations between the chemokine family, including SRC and ASPL-TFE3, might lead to the identification of useful candidates for novel therapeutic targets. These present and previous data suggest that dasatinib might suppress cell growth through CLL4, SRC, *UPP1*, and *ASPSCR1–TFE3*.

Recently, certain clinical trials have reported the efficacy of anti-angiogenic agents including sunitinib and cediranib in ASPS [29, 30, 47]. Sunitinib is an oral, multi-targeted TKI targeting KIT, PDGFRs, VEGFRs, FLT3, and RET [29, 47]. Cediranib is an orally bioavailable inhibitor of all VEGFRs [30]. Therefore, we verified and compared the antitumor activities of five drugs—pazopanib, cabozantinib, dasatinib, cediranib, and sunitinib—in ASPS cell lines (S1 Table). The half maximal inhibitory concentration (IC50) values showed that both cediranib and sunitinib had significantly great antitumor activity against ASPS according to the results of these clinical trials. These present and previous findings suggest that both cediranib and sunitinib may be effective in the treatment of ASPS.

In conclusion, our results showed that cabozantinib and dasatinib exhibited antitumor activity by inhibiting either or both of the growth and invasion of ASPS cells, in part through their respective inhibition of c-MET and SRC. These present and previous data suggest that c-MET is likely the major target in ASPS cells. These findings suggest that cabozantinib may be a useful therapeutic option in patients with ASPS, particularly those with tumors resistant to pazopanib.

## Supporting information

S1 Fig T-PCR of ASPL-TFE3 fusion in ASPS-KY cell line. A: ASPL-TFE3 and B: GAPDH. RNA was extracted from the ASPS-KY cell line and ASPS surgical material using an RNeasy Plus Mini kit (Qiagen, Hilden, Germany), and first-strand synthesis was performed using 5 μg of RNA and the SuperScript® IV First-Strand Synthesis System (Thermo Fisher Scientific, Waltham, MA, USA). We performed RT-PCR analyses to evaluate the expression of ASPL-TFE3 and GAPDH using PCR SuperMix (Thermo Fisher Scientific). The human ASPL-TFE3 primer sequences were as follows: 5’- CCAAGCCAAAGAAGTCCAAG -3’ and 5’- CAAGCAGATTCCCTGACACA -3’. Human GAPDH was used as a loading control, with primers as follows: 5’-GAAGGTGAAGGTCGGAGTC3’ and 5’- GAAGATGGTGATGGGATTT-3’. The ASPL-TFE3 fusion gene was confirmed in the ASPS-KY cell line.
(PPTX)

S2 Fig Effects of cabozantinib on VEGFR2 phosphorylation and a VEGFR2 expression analysis by western blotting in ASPS cell line. We also investigated whether or not cabozantinib inhibits VEGFR2 phosphorylation in ASPS cells. We confirmed that the expression of VEGFR2 phosphorylation stimulated by VEGFA was dose-dependently inhibited by cabozantinib.
(PPTX)

S1 Table IC50 values of TK inhibitors. Pazopanib (GW786034), dasatinib (BMS-354825), and cediranib (S1017) were purchased from Selleck Chemicals (Houston, TX, USA). Cabozantinib (XL-184) was obtained from ChemScene (Monmouth Junction, NJ, USA). Sunitinib (PZ0012) was purchased from Sigma Aldrich (St. Louis, MO, USA). ASPS cells were seeded into 96-well plates at 3000 cells/well. The next day, different concentrations of inhibitors or DMSO (as a vehicle control) were added to each well. After 96 h, the inhibitory effect of these inhibitors on the growth of ASPS cell lines was assessed using an Alamar Blue cell viability assay (Thermo Fisher Scientific). The IC50 was calculated using the GraphPad Prism software program (GraphPad Software, Inc., San Diego, CA, USA).
(PPTX)

## References

[1] Lieberman PH, Brennan MF, Kimmel M, Erlandson RA, Garin-Chesa P, Flehinger BY. Alveolar soft-part sarcoma. A clinico-pathologic study of half a century. Cancer. 1989;63(1):1–13. Epub 1989/01/01. . 2642727 10.1002/1097-0142(19890101)63:1<1::aid-cncr2820630102>3.0.co;2-e

[2] Portera CA Jr., Ho V, Patel SR, Hunt KK, Feig BW, Respondek PM, et al Alveolar soft part sarcoma: clinical course and patterns of metastasis in 70 patients treated at a single institution. Cancer. 2001;91(3):585–91. Epub 2001/02/15. . 11169942 10.1002/1097-0142(20010201)91:3<585::aid-cncr1038>3.0.co;2-0

[3] Ogose A, Yazawa Y, Ueda T, Hotta T, Kawashima H, Hatano H, et al Alveolar soft part sarcoma in Japan: multi-institutional study of 57 patients from the Japanese Musculoskeletal Oncology Group. Oncology. 2003;65(1):7–13. Epub 2003/07/03. doi: 71199 . 12837977 10.1159/000071199

[4] Ogura K, Beppu Y, Chuman H, Yoshida A, Yamamoto N, Sumi M, et al Alveolar soft part sarcoma: a single-center 26-patient case series and review of the literature. Sarcoma. 2012;2012:907179 Epub 2012/06/06. doi: 10.1155/2012/907179 ; PubMed Central PMCID: PMCPMC3362210. 22666000 10.1155/2012/907179 PMC3362210

[5] Casanova M, Ferrari A, Bisogno G, Cecchetto G, Basso E, De Bernardi B, et al Alveolar soft part sarcoma in children and adolescents: A report from the Soft-Tissue Sarcoma Italian Cooperative Group. Ann Oncol. 2000;11(11):1445–9. Epub 2001/01/06. . 11142485 10.1023/a:1026579623136

[6] Pennacchioli E, Fiore M, Collini P, Radaelli S, Dileo P, Stacchiotti S, et al Alveolar soft part sarcoma: clinical presentation, treatment, and outcome in a series of 33 patients at a single institution. Ann Surg Oncol. 2010;17(12):3229–33. Epub 2010/07/02. doi: 10.1245/s10434-010-1186-x . 20593242 10.1245/s10434-010-1186-x

[7] Lazar AJ, Lahat G, Myers SE, Smith KD, Zou C, Wang WL, et al Validation of potential therapeutic targets in alveolar soft part sarcoma: an immunohistochemical study utilizing tissue microarray. Histopathology. 2009;55(6):750–5. Epub 2009/12/17. doi: 10.1111/j.1365-2559.2009.03436.x . 20002771 10.1111/j.1365-2559.2009.03436.x

[8] Hunter T. Tyrosine phosphorylation: thirty years and counting. Curr Opin Cell Biol. 2009;21(2):140–6. Epub 2009/03/10. doi: 10.1016/j.ceb.2009.01.028 ; PubMed Central PMCID: PMCPMC2670436. 19269802 10.1016/j.ceb.2009.01.028 PMC2670436

[9] Robinson DR, Wu YM, Lin SF. The protein tyrosine kinase family of the human genome. Oncogene. 2000;19(49):5548–57. Epub 2000/12/15. doi: 10.1038/sj.onc.1203957 . 11114734 10.1038/sj.onc.1203957

[10] Maemondo M, Inoue A, Kobayashi K, Sugawara S, Oizumi S, Isobe H, et al Gefitinib or chemotherapy for non-small-cell lung cancer with mutated EGFR. The New England journal of medicine. 2010;362(25):2380–8. Epub 2010/06/25. doi: 10.1056/NEJMoa0909530 . 20573926 10.1056/NEJMoa0909530

[11] Demetri GD, von Mehren M, Blanke CD, Van den Abbeele AD, Eisenberg B, Roberts PJ, et al Efficacy and safety of imatinib mesylate in advanced gastrointestinal stromal tumors. The New England journal of medicine. 2002;347(7):472–80. Epub 2002/08/16. doi: 10.1056/NEJMoa020461 . 12181401 10.1056/NEJMoa020461

[12] Slamon D, Eiermann W, Robert N, Pienkowski T, Martin M, Press M, et al Adjuvant trastuzumab in HER2-positive breast cancer. The New England journal of medicine. 2011;365(14):1273–83. Epub 2011/10/14. doi: 10.1056/NEJMoa0910383 ; PubMed Central PMCID: PMC3268553. 21991949 10.1056/NEJMoa0910383 PMC3268553

[13] Christensen JG, Schreck R, Burrows J, Kuruganti P, Chan E, Le P, et al A selective small molecule inhibitor of c-Met kinase inhibits c-Met-dependent phenotypes in vitro and exhibits cytoreductive antitumor activity in vivo. Cancer Res. 2003;63(21):7345–55. Epub 2003/11/13. . 14612533

[14] Laird AD, Cherrington JM. Small molecule tyrosine kinase inhibitors: clinical development of anticancer agents. Expert Opin Investig Drugs. 2003;12(1):51–64. Epub 2003/01/09. doi: 10.1517/13543784.12.1.51 . 12517254 10.1517/13543784.12.1.51

[15] Covell DG, Wallqvist A, Kenney S, Vistica DT. Bioinformatic analysis of patient-derived ASPS gene expressions and ASPL-TFE3 fusion transcript levels identify potential therapeutic targets. PloS one. 2012;7(11):e48023 Epub 2012/12/12. doi: 10.1371/journal.pone.0048023 ; PubMed Central PMCID: PMC3511488. 23226201 10.1371/journal.pone.0048023 PMC3511488

[16] Kenney S, Vistica DT, Stockwin LH, Burkett S, Hollingshead MG, Borgel SD, et al ASPS-1, a novel cell line manifesting key features of alveolar soft part sarcoma. Journal of pediatric hematology/oncology. 2011;33(5):360–8. Epub 2011/05/10. doi: 10.1097/MPH.0b013e3182002f9f . 21552147 10.1097/MPH.0b013e3182002f9f PMC7518051

[17] Kobos R, Nagai M, Tsuda M, Merl MY, Saito T, Lae M, et al Combining integrated genomics and functional genomics to dissect the biology of a cancer-associated, aberrant transcription factor, the ASPSCR1-TFE3 fusion oncoprotein. The Journal of pathology. 2013;229(5):743–54. Epub 2013/01/05. doi: 10.1002/path.4158 ; PubMed Central PMCID: PMC4083568. 23288701 10.1002/path.4158 PMC4083568

[18] Ladanyi M, Lui MY, Antonescu CR, Krause-Boehm A, Meindl A, Argani P, et al The der(17)t(X;17)(p11;q25) of human alveolar soft part sarcoma fuses the TFE3 transcription factor gene to ASPL, a novel gene at 17q25. Oncogene. 2001;20(1):48–57. Epub 2001/03/13. doi: 10.1038/sj.onc.1204074 . 11244503 10.1038/sj.onc.1204074

[19] Stockwin LH, Vistica DT, Kenney S, Schrump DS, Butcher DO, Raffeld M, et al Gene expression profiling of alveolar soft-part sarcoma (ASPS). BMC cancer. 2009;9:22 Epub 2009/01/17. doi: 10.1186/1471-2407-9-22 ; PubMed Central PMCID: PMCPMC2635365. 19146682 10.1186/1471-2407-9-22 PMC2635365

[20] Tsuda M, Davis IJ, Argani P, Shukla N, McGill GG, Nagai M, et al TFE3 fusions activate MET signaling by transcriptional up-regulation, defining another class of tumors as candidates for therapeutic MET inhibition. Cancer Res. 2007;67(3):919–29. Epub 2007/02/07. doi: 10.1158/0008-5472.CAN-06-2855 . 17283122 10.1158/0008-5472.CAN-06-2855

[21] Stacchiotti S, Tamborini E, Marrari A, Brich S, Rota SA, Orsenigo M, et al Response to sunitinib malate in advanced alveolar soft part sarcoma. Clin Cancer Res. 2009;15(3):1096–104. Epub 2009/02/04. doi: 10.1158/1078-0432.CCR-08-2050 . 19188185 10.1158/1078-0432.CCR-08-2050

[22] Lazar AJ, Das P, Tuvin D, Korchin B, Zhu Q, Jin Z, et al Angiogenesis-promoting gene patterns in alveolar soft part sarcoma. Clin Cancer Res. 2007;13(24):7314–21. Epub 2007/12/21. doi: 10.1158/1078-0432.CCR-07-0174 . 18094412 10.1158/1078-0432.CCR-07-0174

[23] Ranieri G, Mammi M, Donato Di Paola E, Russo E, Gallelli L, Citraro R, et al Pazopanib a tyrosine kinase inhibitor with strong anti-angiogenetic activity: a new treatment for metastatic soft tissue sarcoma. Crit Rev Oncol Hematol. 2014;89(2):322–9. Epub 2013/09/18. doi: 10.1016/j.critrevonc.2013.08.012 . 24041629 10.1016/j.critrevonc.2013.08.012

[24] Funakoshi Y, Okada M, Kawata S, Ito N, Abe K, Moriuchi H. The Significant Effects of Pazopanib on Multiple Pulmonary Metastatic Lesions of Alveolar Soft Part Sarcoma: A Case Report. Journal of pediatric hematology/oncology. 2017 Epub 2017/01/07. doi: 10.1097/MPH.0000000000000736 . 28060129 10.1097/MPH.0000000000000736

[25] Nakamura T, Matsumine A, Kawai A, Araki N, Goto T, Yonemoto T, et al The clinical outcome of pazopanib treatment in Japanese patients with relapsed soft tissue sarcoma: A Japanese Musculoskeletal Oncology Group (JMOG) study. Cancer. 2016;122(9):1408–16. Epub 2016/03/13. doi: 10.1002/cncr.29961 ; PubMed Central PMCID: PMC5069581. 26970174 10.1002/cncr.29961 PMC5069581

[26] Read WL, Williams F. Metastatic Alveolar Soft Part Sarcoma Responsive to Pazopanib after Progression through Sunitinib and Bevacizumab: Two Cases. Case reports in oncology. 2016;9(3):639–43. Epub 2016/12/07. doi: 10.1159/000450545 ; PubMed Central PMCID: PMC5118836. 27920695 10.1159/000450545 PMC5118836

[27] Wagner AJ, Goldberg JM, Dubois SG, Choy E, Rosen L, Pappo A, et al Tivantinib (ARQ 197), a selective inhibitor of MET, in patients with microphthalmia transcription factor-associated tumors: results of a multicenter phase 2 trial. Cancer. 2012;118(23):5894–902. Epub 2012/05/19. doi: 10.1002/cncr.27582 . 22605650 10.1002/cncr.27582

[28] Azizi AA, Haberler C, Czech T, Gupper A, Prayer D, Breitschopf H, et al Vascular-endothelial-growth-factor (VEGF) expression and possible response to angiogenesis inhibitor bevacizumab in metastatic alveolar soft part sarcoma. The Lancet Oncology. 2006;7(6):521–3. Epub 2006/06/06. doi: 10.1016/S1470-2045(06)70729-X . 16750504 10.1016/S1470-2045(06)70729-X

[29] Stacchiotti S, Negri T, Zaffaroni N, Palassini E, Morosi C, Brich S, et al Sunitinib in advanced alveolar soft part sarcoma: evidence of a direct antitumor effect. Ann Oncol. 2011;22(7):1682–90. Epub 2011/01/19. doi: 10.1093/annonc/mdq644 . 21242589 10.1093/annonc/mdq644

[30] Kummar S, Allen D, Monks A, Polley EC, Hose CD, Ivy SP, et al Cediranib for metastatic alveolar soft part sarcoma. Journal of clinical oncology: official journal of the American Society of Clinical Oncology. 2013;31(18):2296–302. Epub 2013/05/01. doi: 10.1200/jco.2012.47.4288 ; PubMed Central PMCID: PMCPMC3677840. 23630200 10.1200/JCO.2012.47.4288 PMC3677840

[31] Goldberg JM, Gavcovich T, Saigal G, Goldman JW, Rosen LS. Extended progression-free survival in two patients with alveolar soft part sarcoma exposed to tivantinib. Journal of clinical oncology: official journal of the American Society of Clinical Oncology. 2014;32(34):e114–6. Epub 2014/02/20. doi: 10.1200/JCO.2013.48.7462 ; PubMed Central PMCID: PMC4876351. 24550414 10.1200/JCO.2013.48.7462 PMC4876351

[32] Li T, Wang L, Wang H, Zhang S, Atikan K, Zhang X, et al A retrospective analysis of 14 consecutive Chinese patients with unresectable or metastatic alveolar soft part sarcoma treated with sunitinib. Investigational new drugs. 2016;34(6):701–6. Epub 2016/09/09. doi: 10.1007/s10637-016-0390-3 . 27604635 10.1007/s10637-016-0390-3

[33] Araujo JC, Trudel GC, Saad F, Armstrong AJ, Yu EY, Bellmunt J, et al Docetaxel and dasatinib or placebo in men with metastatic castration-resistant prostate cancer (READY): a randomised, double-blind phase 3 trial. The Lancet Oncology. 2013;14(13):1307–16. Epub 2013/11/12. doi: 10.1016/S1470-2045(13)70479-0 . 24211163 10.1016/S1470-2045(13)70479-0 PMC5478530

[34] Grullich C. Cabozantinib: a MET, RET, and VEGFR2 tyrosine kinase inhibitor. Recent results in cancer research Fortschritte der Krebsforschung Progres dans les recherches sur le cancer. 2014;201:207–14. Epub 2014/04/24. doi: 10.1007/978-3-642-54490-3_12 . 24756794 10.1007/978-3-642-54490-3_12

[35] Talpaz M, Shah NP, Kantarjian H, Donato N, Nicoll J, Paquette R, et al Dasatinib in imatinib-resistant Philadelphia chromosome-positive leukemias. The New England journal of medicine. 2006;354(24):2531–41. Epub 2006/06/16. doi: 10.1056/NEJMoa055229 . 16775234 10.1056/NEJMoa055229

[36] Yakes FM, Chen J, Tan J, Yamaguchi K, Shi Y, Yu P, et al Cabozantinib (XL184), a novel MET and VEGFR2 inhibitor, simultaneously suppresses metastasis, angiogenesis, and tumor growth. Molecular cancer therapeutics. 2011;10(12):2298–308. Epub 2011/09/20. doi: 10.1158/1535-7163.MCT-11-0264 . 21926191 10.1158/1535-7163.MCT-11-0264

[37] Maslikowski BM, Neel BD, Wu Y, Wang L, Rodrigues NA, Gillet G, et al Cellular processes of v-Src transformation revealed by gene profiling of primary cells—implications for human cancer. BMC cancer. 2010;10:41 Epub 2010/02/16. doi: 10.1186/1471-2407-10-41 ; PubMed Central PMCID: PMC2837010. 20152043 10.1186/1471-2407-10-41 PMC2837010

[38] Kim Y, Kobayashi E, Kubota D, Suehara Y, Mukaihara K, Akaike K, et al Reduced argininosuccinate synthetase expression in refractory sarcomas: Impacts on therapeutic potential and drug resistance. Oncotarget. 2016;7(43):70832–44. Epub 2016/09/30. doi: 10.18632/oncotarget.12225 . 27683125 10.18632/oncotarget.12225 PMC5342592

[39] Hoshino M, Ogose A, Kawashima H, Izumi T, Hotta T, Hatano H, et al Molecular analyses of cell origin and detection of circulating tumor cells in the peripheral blood in alveolar soft part sarcoma. Cancer genetics and cytogenetics. 2009;190(2):75–80. Epub 2009/04/22. doi: 10.1016/j.cancergencyto.2008.11.014 . 19380023 10.1016/j.cancergencyto.2008.11.014

[40] Nakayama R, Zhang YX, Czaplinski JT, Anatone AJ, Sicinska ET, Fletcher JA, et al Preclinical activity of selinexor, an inhibitor of XPO1, in sarcoma. Oncotarget. 2016;7(13):16581–92. Epub 2016/02/27. doi: 10.18632/oncotarget.7667 ; PubMed Central PMCID: PMC4941336. 26918731 10.18632/oncotarget.7667 PMC4941336

[41] You WK, Sennino B, Williamson CW, Falcon B, Hashizume H, Yao LC, et al VEGF and c-Met blockade amplify angiogenesis inhibition in pancreatic islet cancer. Cancer Res. 2011;71(14):4758–68. Epub 2011/05/27. doi: 10.1158/0008-5472.CAN-10-2527 ; PubMed Central PMCID: PMCPMC3138890. 21613405 10.1158/0008-5472.CAN-10-2527 PMC3138890

[42] Jun HJ, Lee J, Lim do H, Park JO, Ahn G, Seo SW, et al Expression of MET in alveolar soft part sarcoma. Medical oncology (Northwood, London, England). 2010;27(2):459–65. Epub 2009/05/28. doi: 10.1007/s12032-009-9234-8 . 19472090 10.1007/s12032-009-9234-8

[43] Gao CF, Vande Woude GF. HGF/SF-Met signaling in tumor progression. Cell Res. 2005;15(1):49–51. Epub 2005/02/03. doi: 10.1038/sj.cr.7290264 . 15686627 10.1038/sj.cr.7290264

[44] Munshi N, Jeay S, Li Y, Chen CR, France DS, Ashwell MA, et al ARQ 197, a novel and selective inhibitor of the human c-Met receptor tyrosine kinase with antitumor activity. Molecular cancer therapeutics. 2010;9(6):1544–53. Epub 2010/05/21. doi: 10.1158/1535-7163.MCT-09-1173 . 20484018 10.1158/1535-7163.MCT-09-1173

[45] Basilico C, Pennacchietti S, Vigna E, Chiriaco C, Arena S, Bardelli A, et al Tivantinib (ARQ197) displays cytotoxic activity that is independent of its ability to bind MET. Clinical cancer research: an official journal of the American Association for Cancer Research. 2013;19(9):2381–92. Epub 2013/03/28. doi: 10.1158/1078-0432.CCR-12-3459 . 23532890 10.1158/1078-0432.CCR-12-3459

[46] Katayama R, Aoyama A, Yamori T, Qi J, Oh-hara T, Song Y, et al Cytotoxic activity of tivantinib (ARQ 197) is not due solely to c-MET inhibition. Cancer research. 2013;73(10):3087–96. Epub 2013/04/20. doi: 10.1158/0008-5472.CAN-12-3256 ; PubMed Central PMCID: PMC3759033. 23598276 10.1158/0008-5472.CAN-12-3256 PMC3759033

[47] Hindi N PS, Maestro R, Dei Tos AP, Palassini E, Morosi C, Messina A, Provenzano S, Negri T, Fiore M, Gronchi A, Brenca M, Casali PG, Stacchiotti S. Sunitinib malate in advanced alveolar soft part sarcoma (ASPS): A final update after the closure of the named use program. Journal of clinical oncology: official journal of the American Society of Clinical Oncology. 2015;33:(suppl; abstr 10562).

