# Supplementary material for: Cabozantinib and dastinib exert anti-tumor activity in alveolar soft part sarcoma
Source: PLoS One. 2017 Sep 25;12(9):e0185321. doi: 10.1371/journal.pone.0185321 (PMC5612696; doi:10.1371/journal.pone.0185321)
Supplement: S1 Fig — A: ASPL-TFE3 and B: GAPDH. RNA was extracted from the ASPS-KY cell line and ASPS surgical material using an RNeasy Plus Mini kit (Qiagen, Hilden, Germany), and first-strand synthesis was performed using 5 μg of RNA and the SuperScript® IV First-Strand Synthesis System (Thermo Fisher Scientific, Waltham, MA, USA). We performed RT-PCR analyses to evaluate the expression of ASPL-TFE3 and GAPDH using PCR SuperMix (Thermo Fisher Scientific). The human ASPL-TFE3 primer sequences were as follows: 5’- CCAAGCCAAAGAAGTCCAAG -3’ and 5’- CAAGCAGATTCCCTGACACA -3’. Human GAPDH was used as a loading control, with primers as follows: 5’-GAAGGTGAAGGTCGGAGTC3’ and 5’- GAAGATGGTGATGGGATTT-3’. The ASPL-TFE3 fusion gene was confirmed in the ASPS-KY cell line. (PPTX) [file pone.0185321.s001.pptx]

## Slide 1
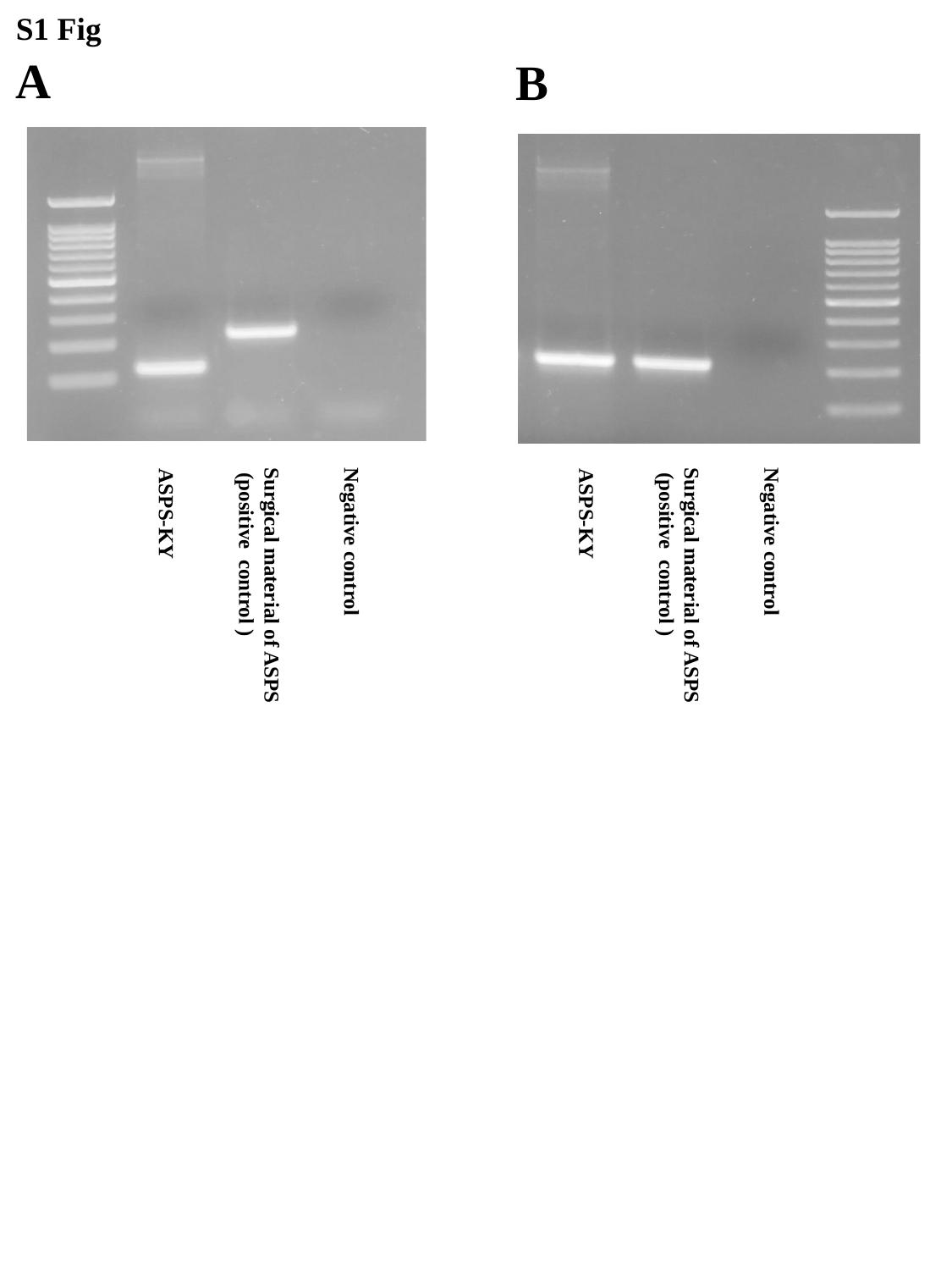

S1 Fig
A
B
ASPS-KY
ASPS-KY
Negative control
Negative control
Surgical material of ASPS
 (positive control )
Surgical material of ASPS
 (positive control )
