# Supplementary figures and images for: Cabozantinib and dastinib exert anti-tumor activity in alveolar soft part sarcoma
Source: PLoS One. 2017 Sep 25;12(9):e0185321. doi: 10.1371/journal.pone.0185321 (PMC5612696; doi:10.1371/journal.pone.0185321)

## Slide 1
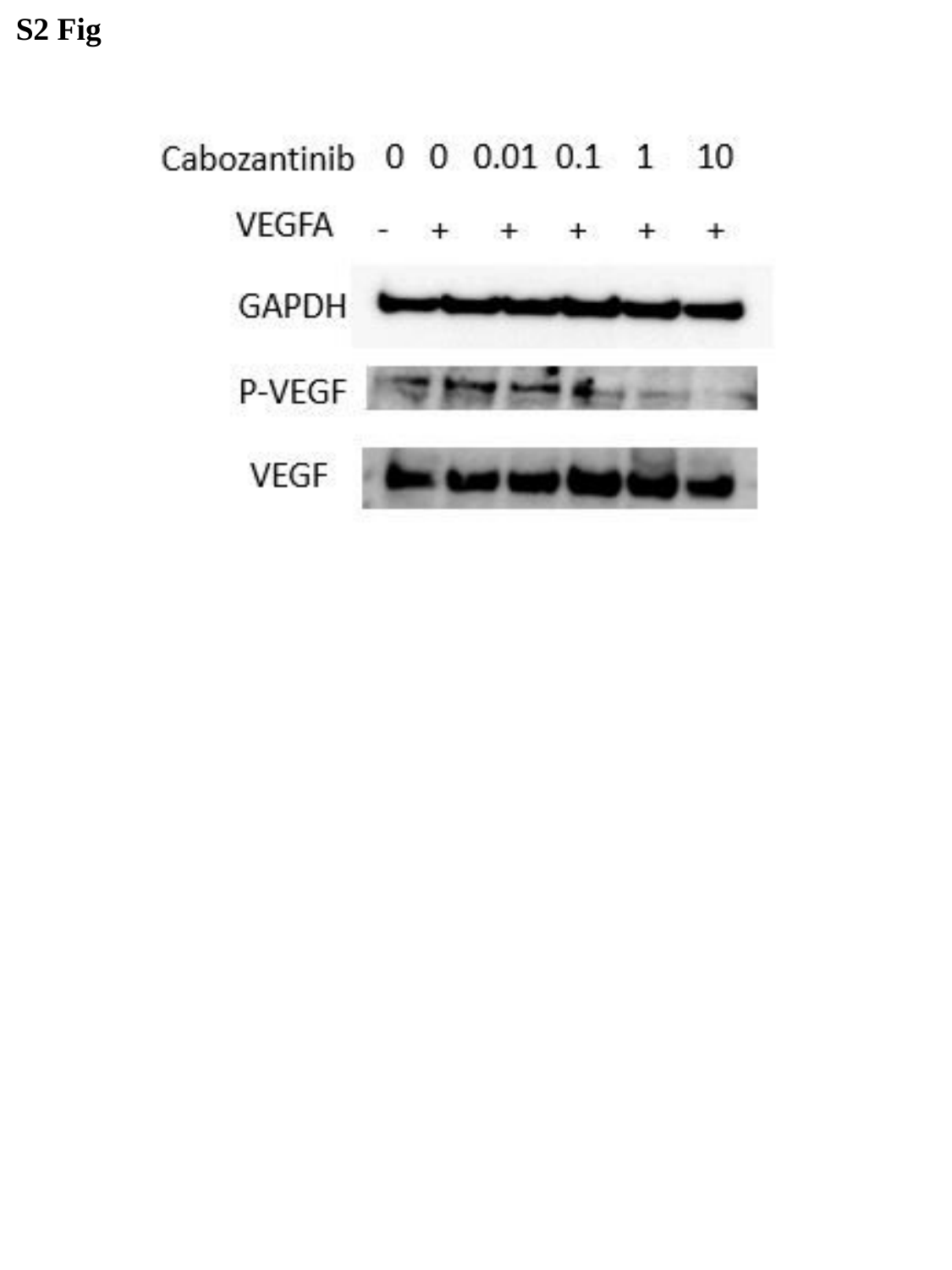

S2 Fig

Supplement: S2 Fig — We also investigated whether or not cabozantinib inhibits VEGFR2 phosphorylation in ASPS cells. We confirmed that the expression of VEGFR2 phosphorylation stimulated by VEGFA was dose-dependently inhibited by cabozantinib. (PPTX) [file pone.0185321.s002.pptx]

## Slide 1
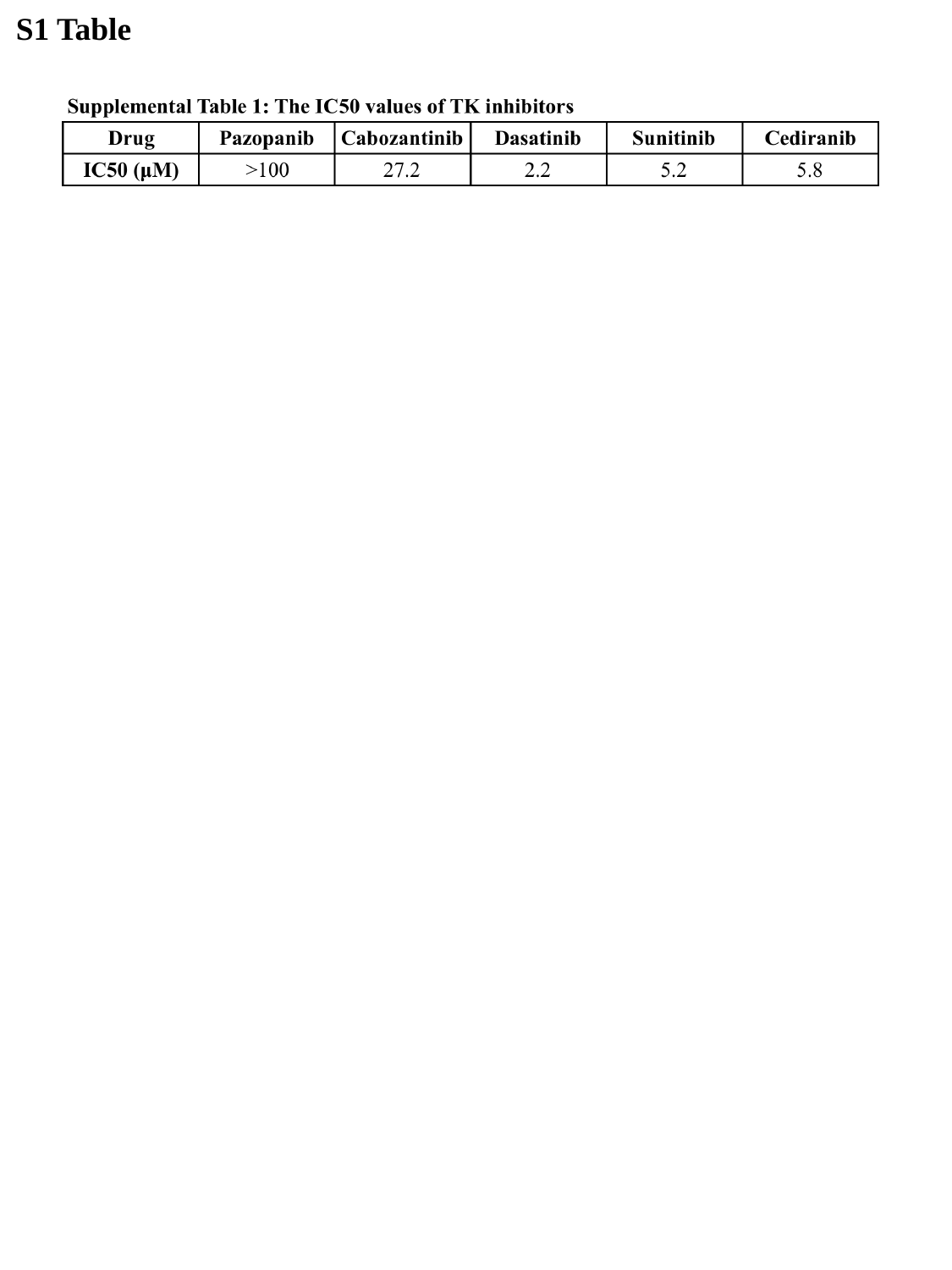

S1 Table

Supplement: S1 Table — Pazopanib (GW786034), dasatinib (BMS-354825), and cediranib (S1017) were purchased from Selleck Chemicals (Houston, TX, USA). Cabozantinib (XL-184) was obtained from ChemScene (Monmouth Junction, NJ, USA). Sunitinib (PZ0012) was purchased from Sigma Aldrich (St. Louis, MO, USA). ASPS cells were seeded into 96-well plates at 3000 cells/well. The next day, different concentrations of inhibitors or DMSO (as a vehicle control) were added to each well. After 96 h, the inhibitory effect of these inhibitors on the growth of ASPS cell lines was assessed using an Alamar Blue cell viability assay (Thermo Fisher Scientific). The IC50 was calculated using the GraphPad Prism software program (GraphPad Software, Inc., San Diego, CA, USA). (PPTX) [file pone.0185321.s003.pptx]
